# Clinical Effectiveness, Safety, and Compliance of Two Compounded Formulations of Tacrolimus Eye Drops: An Open-Label, Sequential Prospective Study

**DOI:** 10.3390/ijms25189847

**Published:** 2024-09-12

**Authors:** María Puente-Iglesias, Andrea Cuartero-Martínez, Rosario Touriño-Peralba, María Teresa Rodríguez-Ares, María Jesús Giráldez, Eva Yebra-Pimentel, Laura García-Quintanilla, Xurxo García-Otero, Miguel González-Barcia, Irene Zarra-Ferro, Francisco J. Otero-Espinar, Anxo Fernández-Ferreiro, Ana Castro-Balado

**Affiliations:** 1 Pharmacy Department, University Clinical Hospital of Santiago de Compostela (SERGAS), 15706 Santiago de Compostela, Spainandrea.cuartero@rai.usc.es (A.C.-M.);; 2 FarmaCHUSLab Group, Health Research Institute of Santiago de Compostela (IDIS), 15706 Santiago de Compostela, Spain; 3 Department of Pharmacology, Pharmacy and Pharmaceutical Technology, Faculty of Pharmacy, University of Santiago de Compostela (USC), 15782 Santiago de Compostela, Spainrancisco.otero@usc.es (F.J.O.-E.); 4 Ophthalmology Department, University Clinical Hospital of Santiago de Compostela (SERGAS), 15706 Santiago de Compostela, Spain; rtourinop2@gmail.com (R.T.-P.);; 5 Department of Applied Physics, Optometry, Faculty of Optics and Optometry, University of Santiago de Compostela (USC), 15782 Santiago de Compostela, Spain; mjesus.giraldez@usc.es (M.J.G.);; 6 Molecular Imaging Biomarkers and Theragnosis Lab Group, Center for Research in Molecular Medicine and Chronic Diseases (CiMUS), University of Santiago de Compostela (USC), 15782 Santiago de Compostela, Spain

**Keywords:** tacrolimus, hydroxypropyl-β-cyclodextrin, eye drops, efficacy, safety

## Abstract

Ophthalmic tacrolimus compounded formulations are usually made from the commercial intravenous presentation, which contains ethanol as a solubilizer due to the low solubility of tacrolimus. The use of cyclodextrins is presented as an alternative to ethanol, an ocular irritant excipient, to avoid its long-term irritant effects. Open-label, sequential, prospective study to compare effectiveness, safety, and adherence of a new formulation of 0.015% tacrolimus with cyclodextrins (TCD) versus 0.03% tacrolimus with ethanol (TE). The ocular evaluation was assessed by ocular signs, corneal staining, subjective questionnaires as Visual Function Questionnaire (VFQ-25) and Visual Analogue Scale (VAS) of symptoms, lacrimal stability, ocular redness, and intraocular pressure. Compliance was assessed by VAS of adherence and empirically (difference between theoretical and actual consumption). Clinical ocular signs and corneal staining score remained stable for most patients 3 months after switching formulations. The TCD formulation did not modify the tear stability and intraocular pressure of the treated patients compared to the TE formulation. TCD eye drops significantly decreased the subjective pain values on VFQ-25 scale and burning sensation on the VAS symptom scale in comparison to TE formulation after 3 months after the change to TCD formulation. The novel tacrolimus in cyclodextrins formulation is a promising alternative for treating inflammatory ocular pathologies refractory to first-line treatments.

## 1. Introduction

Tacrolimus is a cyclic macrolide lactone with immunosuppressive effect which inhibits calcineurin FK506, causing a decrease in the transcription of proinflammatory cytokines. It is commonly used as a prophylactic treatment for organ rejection in solid transplant recipients and for the treatment of allograft rejection resistant to other immunosuppressive drugs [1]. Off-label clinical indications for tacrolimus include the treatment of autoimmune diseases such as atopic dermatitis or psoriasis, among others [2,3]. In ophthalmic pathologies, the use of off-label tacrolimus has been successful in the prevention of corneal rejection, dry eye syndrome, vernal keratoconjunctivitis, atopic keratoconjunctivitis, corneal and conjunctival immune diseases [3,4,5,6]. In these ocular pathologies, inflammation has been shown to be a key factor in the pathogenesis, and their treatment include topical corticosteroids, mast cell stabilizers, antihistaminic and immunomodulatory agents [7]. Therapeutic alternatives, such as immunosuppressive drugs as cyclosporine and tacrolimus, are necessary in refractory cases [8].

Tacrolimus eye drops are not commercially available, so they are usually elaborated as a compounded formulation in Hospital Pharmacy Departments (HPDs). The limiting factor in their elaboration is the low water solubility of tacrolimus [9]. One of the most widespread compounded formulation is made by reformulating the intravenous presentation (Prograf^®^), where tacrolimus is solubilized using ethanol, an irritant excipient for the ocular surface [10,11]. Ophthalmic use of ethanol has been associated with uncomfortable ocular application that can affect adherence, as well as patient safety due to its toxicity when used by this route [12]. Different alternatives have been developed to avoid the use of ethanol in tacrolimus eye drops, but the complexity of the preparation and the need for specific equipment make clinical translation to HPDs difficult [13,14,15]. Against this background, our group developed an alternative compounded formulation using 2-HydroxyPropil-Beta-Cyclodextrin (HPβCD) as solubilizer [16]. According to the European Medicines Agency (EMA), HPβCD is the safest and most suitable cyclodextrin for use in topical ophthalmic applications [17]. García-Otero et al. developed and characterized the solubility, stability and mucoadhesive properties of this new formulation [16]. The addition of HPβCD resulted in an increase in the solubility and stability of tacrolimus, accompanied by a notable change in the solution’s viscosity and adhesiveness. This formulation has been also evaluated in animal models of inflammation, achieving a reduction in leukocytes and cytokines at the ocular level [18]. The new formulation, with greater ocular biopermanence, allows for a reduction in tacrolimus concentration, achieving the desired therapeutic effect and, at the same time, reducing the potential risk of mutagenicity when tacrolimus is used for prolonged periods [19,20].

The elaboration of the new formulation with cyclodextrins was transferred to the HPD in response to demand from prescribing ophthalmologists, with the objective of improving patient safety while maintaining the efficacy. The aim of this study is to evaluate and compare the effectivity, safety, and compliance of 0.015% tacrolimus eye drops with HPβCD (TCD) and the previously used 0.03% tacrolimus eye drops with ethanol (TE) elaborated from Prograf^®^ in the management of ocular inflammatory pathologies.

## 2. Results

### 2.1. Population and Treatment Characteristics

The switch to the TCD formulation was made in agreement with the Ophthalmology Department for a total of 56 patients. The median age was 52.5 years (Q_1_–Q_3_: 18–66), with 25.0% under 18 years old and 16.1% over 70 years old. The proportion of men was slightly higher (64.0%). In terms of diagnosis, tacrolimus was administered to 39.3% of patients for the treatment of conjunctivitis/atopy, 37.5% for prophylaxis/treatment of rejection after corneal transplant, and 9.0% for the treatment of Sjögren’s syndrome. The remaining 14.2% of patients suffered from rare diseases such as Lyell or Thyggesson syndrome, or caustic burns.

**Figure 1:**
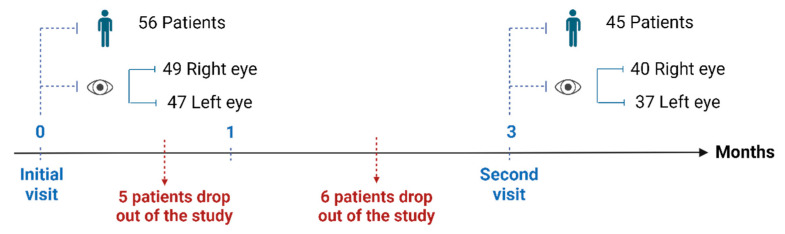
Patient flow and studied eyes during the two study visits. Created in BioRender.

Patients were instructed to apply the eye drops in the affected eye. However, a considerable proportion of patients exhibited biocular involvement, leading them to apply the eye drops to both eyes. The dosage prior to the start of the study varied between one drop in both eyes (Oculus Uterque, OU) every 6 h and one drop in OU every 48 h, according to the pathology and patient requirements. All patients used artificial tears, 21.4% of patients also received treatment with corticosteroids and 9.0% of patients were treated with autologous serum. The average number of concomitant eye drops prescribed was 2. Between the initial and second visit, 11 patients abandoned treatment (Figure 1), five of them did so before the first month of treatment due to conjunctival hyperemia and a sticky sensation with difficulty blinking after application. In these patients, TE eye drops were reintroduced. The remaining six patients returned to TE eye drops because they had become tolerant to the burning sensation and reported a more satisfactory application due to the lower viscosity of TE eye drops. A total of 9 patients had to change the dosage after switching eye drops: 3 patients needed to increase the dose and 6 had to decrease it. The percentage of patients receiving concomitant corticosteroid treatment decreased from 21.4% to 13.3% after switching from TE to TCD.

### 2.2. Clinical Evaluation

For the analysis of clinical ocular parameters, differences between visits were evaluated. Thus, Best Corrected Visual Acuity (BCVA) was analyzed in 69 eyes of which 27.54% had equal BCVA in both visits and the 44.93% had higher BCVA at visit 2 in comparison with visit 1. The remaining 27.54% of the eyes presented a decreased in BCVA with a worsening of only one optotype in most cases (Table 1). The difference between visits in conjunctival integrity, presence of papillae, tranta points on the corneal limbus and corneal integrity were analyzed in 76 eyes (Table 1). In conjunctiva analyses, most eyes had the same (56.58%) or a lower grade (17.11%) at visit 2 compared to the initial one, while 26.32% had a worsening at visit 2. In the papillae evaluation, 63.13% showed no differences between visits, 9.21% showed an improvement and 27.63% worsened. There were no signs of tranta spots in the limbus in any of the patients evaluated. Finally, in the evaluation of corneal signs it was found that 81.58% of the patients obtained the same value at visit 2 compared to the initial visit, while 18.42% improved the integrity of the corneal signs analyzed. No patient had active corneal ulcerations or erosions during the study.

For corneal staining, the difference between visits on the Oxford scale was analyzed (Table 1). Taking this into account, 72.37% of the eyes had no differences between visits. Within this group, 96.0% were classified as grade 0 at both visits, i.e., they had no corneal staining during the study. A decrease in corneal staining was observed in 25.0% of the eyes analyzed while only 2.63% of the eyes showed an increase in corneal staining. In addition, no symptoms of corneal rejection have been observed in patients who underwent keratoplasty or recurrence of previous pathologies after the change of formulation.

Measurements of Tear Meniscus Height (TMH), Noninvasive Keratograph Break Up Time (NIKBUT) and Ocular Redness (OR) were performed at both visits in the eyes treated with tacrolimus eye drops (Table 2). For the lacrimal stability study, 73 eyes were analyzed in the TMH analysis, of which 54.79% obtained an improvement in the TMH at visit 2, although no significant differences were found. Regarding to NIKBUT analysis, no significant differences were found between tear breakup time measured at each visit. THM and NIKBUT measures of each patient at both visits are shown in Tables S6 and S7 of the Supplementary Material, respectively.

The degree of ocular redness in the bulbar, limbal, and total conjunctiva of 63 eyes was analyzed. In the comparative of OR of the Temporal Bulbar (TB) conjunctiva, Temporal Limbal (TL) and Nasal Limbal (NL), a significant increase in redness was found at visit 2 compared to visit 1 (p_TB_ = 0.0168; p_TL_ = 0.0294; p_NL_ = 0.0182) (Table 2). The comparison in Nasal Bulbar (NB) conjunctiva showed a non-significant difference in OR at visit 2 (*p* = 0.2199). Finally, in the analysis of average OR measured, non-significant differences were found between visits (*p* = 0.0829). Descriptive analysis and the multiple comparison between visits of OR are detailed in Table 2. Thus, no significant differences in intraocular pressure (IOP) were found between visit 1 (median = 14.0 mmHg; Q_1_–Q_3_: 12.0–16.0) and visit 2 (median = 14.0 mmHg; Q_1_–Q_3_: 12.0–15.75) (*p* = 0.7589) in 56 evaluated eyes (Table 2).

### 2.3. Patient Reported Outcomes and Compliance

#### 2.3.1. Reported Outcomes

**Figure 2:**
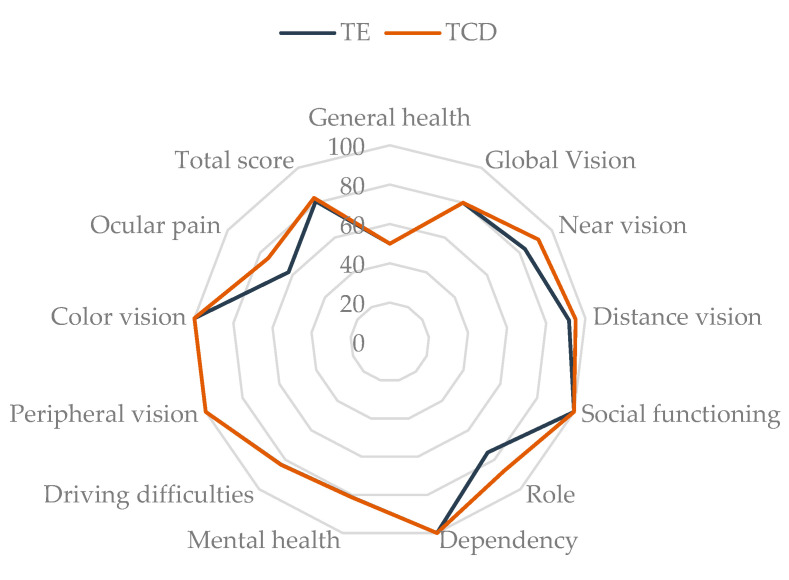
A radial chart showing the subscales examined in the VFQ-25 questionnaire and the medians obtained for TE and TCD. TCD: tacrolimus 0.015% in cyclodextrin; TE: tacrolimus 0.03% prepared from commercial intravenous presentation Prograf^®^.

There was a marked increase in the total score on the Visual Function Questionnaire (VFQ-25), with a median of 80.9 (Q_1_–Q_3_: 66.7–89.7) for TE and 82.8 (Q_1_–Q_3_: 76.6–92.0) for TCD (*p* = 0.003), with higher values being associated with more positive results. In the domain-specific analysis, there was a significant improvement in referred pain when switching to the TCD formulation, with a median punctuation of 62.5 (Q_1_–Q_3_: 56.2–87.5) with TE vs. 75.0 (Q_1_–Q_3_: 62.5–87.5) with TCD (*p* = 0.004). The disparity in this variable is evident in Figure 2, which depicts a redial graph representing the total medians obtained for total score and each subcategory studied in the VFQ-25 questionnaire on a scale from 0 to 100. Of the 12 domains assessed, improvement was evident in four of them with the new formulation (near vision, distance vision, role, ocular pain). Eight domains (general health, global vision, social functioning, dependency, mental health, driving difficulties, peripheral vision, color vision) had the same median score, and none of them had a lower score with the initiation of TCD treatment (Table S8 of the Supplementary Material).

VFQ-25 median total scores were also evaluated based on age groups (<18 years, 18–65 years and >65 years), and a clear age dependence was observed, as shown in Table S9 of the Supplementary Material. The analysis concluded that there is a moderately high negative correlation between age and the VFQ score for TE and TCD (r = −0.647, *p* = 0.001 for TE, r = −0.599, *p* = 0.001 for TCD). A comparison of the medians of the age groups revealed a significant difference in the quality of life between the age groups. As age increases, so does the visual quality of life (*p* = 0.001 for TE, *p* = 0.001 for TCD). It is important to note that the group of patients over the age 65 years showed a mean increase in quality of life of almost 10 points with TCD eye drops.

**Figure 3:**
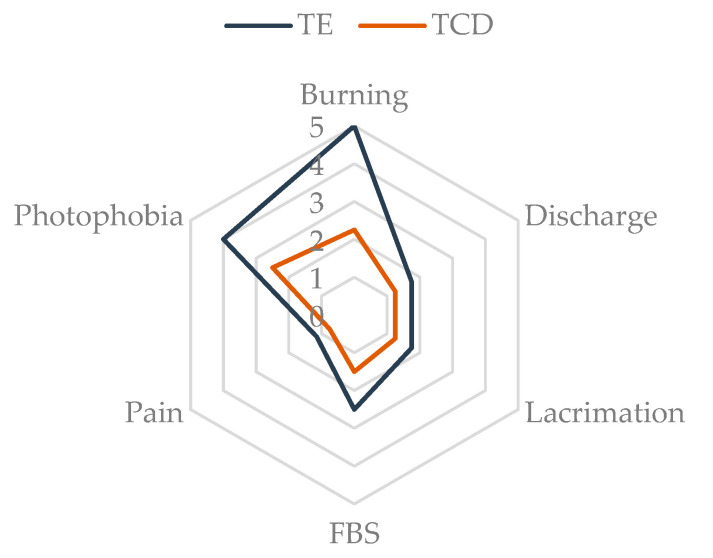
Radial plot of median right and left eye scores for the 6 symptoms examined in the VAS assessment. FBS: foreign body sensation. TCD: tacrolimus 0.015% in cyclodextrin; TE: tacrolimus 0.03% prepared from commercial intravenous presentation Prograf^®^.

Regarding the Visual Analogue Scale (VAS) of symptoms, the improvement in burning was statistically significant in both eyes with a decrease from 5.0 (Q_1_–Q_3_: 1.9–7.0) to 2.0 (Q_1_–Q_3_: 1.0–3.5) (*p* = 0.004) in the right eye, and from 5.0 (Q_1_–Q_3_: 2.0–7.0) to 2.5 (Q_1_–Q_3_: 0.8–4.1) (*p* = 0.003) in the left eye. An improvement in pain perception was only observed in the left eye with a total mean score from 1.3 (Q_1_–Q_3_: 0.0–3.0) to 0.5 (Q_1_–Q_3_: 0.0–2.0) (*p* = 0.03). Table S10 of the Supplementary Material shows the comparison of the median scores obtained at the initial visit with TE and at the 3-month follow-up visit with TCD. In the VAS assessment, the 6 symptoms assessed had a lower score for TCD, as shown in the radial graph in Figure 3. This figure shows mean values for both eyes, which are lower for TCD than TE. The greatest differences are observed in the symptoms of burning and photophobia.

Changes on the VAS symptom scale are shown in Table 3, the percentage of patients reporting improved, worsened, or unchanged symptoms after switching to TCD. An improvement in burning was reported by 51.6% of patients, with 48.5% reducing the intensity of the burning sensation by two or more points on the intensity scale. There was no symptom for which the percentage of worsening was greater than the percentage of improvement.

#### 2.3.2. Compliance

Patient-reported adherence was measured using the VAS adherence scale. Comparing the scales at visit 1 (TE) and visit 2 (TCD), an increase in adherence was observed in 34.3% of patients. It should be noted that 56.6% of the remaining patients continued compliant. Adherence increased significantly from 100.0% (Q_1_–Q_3_: 80–100) for TE to 100.0% (Q_1_–Q_3_: 90–100) for TCD (*p* = 0.003). No association was found between adherence and complexity of dosing. 

In the study cohort, only 44 subjects complied with the instructions and returned two TCD bottles. Consequently, the total number of bottles weighed were 88. In terms of the objective measure of adherence, it was concluded that only 23.6% of patients had a difference between actual and theoretical weight of less than 35% in the two returned containers. No correlation was found between actual and subjective adherence.

## 3. Discussion

Previous studies have analyzed the therapeutic effect of ophthalmic presentations of tacrolimus at different concentrations for different pathologies involving ocular inflammation such as dry eye [21,22,23], vernal keratoconjunctivitis (VKC) [24,25], shield ulcer and corneal epitheliopathy [26], Sjögren’s syndrome dry eye [27], corneal endothelial rejection [28], keratoplastics [29], Thygeson Superficial Punctate Keratitis [30], stromal herpetic keratitis [31]. In the present work, the effectiveness of a tacrolimus 0.015% with cyclodextrins eye drops and how the decrease in concentration could affect in the effectiveness were analyzed using visual acuity to screen for certain ocular pathologies [32], so a positive or neutral variation may demonstrate that there are no negative changes in these patients’ condition. Regarding visual acuity analysis, most of the patients showed equal or improved measures. In relation to the clinical signs analyzed, no significant negative changes were observed in most patients after the change of formulation and the cornea was not affected either by alterations in corneal staining or by other structural changes such as stromal edema. These results indicate that the decrease in tacrolimus concentration does not affect the effectiveness of the treatment.

Tacrolimus formulations with concentrations ranging from 0.03 to 0.1% are the most frequently used in clinical practice. Our original formulation (TE) contained 0.03% tacrolimus, which was subsequently replaced with a 0.015% tacrolimus formulation in HPβCD (TCD). This reduction in concentration was made possible by the increased ocular biopermanence provided by cyclodextrins. The new formulation exhibits comparable effectiveness to the previous one, while receiving 50% less immunosuppressant dose. The lower concentration of immunosuppressant implies greater safety of the formulation, given that greater cytotoxic effects of tacrolimus have been reported at higher concentrations. Sella R. et al. reported in a comparative study in human corneal epithelial cell models that cell survival increased as tacrolimus concentration decreased [19]. Despite the absence of a direct causal relationship between topical tacrolimus treatment and the onset of neoplasia in the ophthalmic region, several instances of conjunctival melanoma have been observed in patients who have undergone tacrolimus eye drop therapy (0.03%). It would be beneficial to investigate this potential correlation [20]. However, it is noteworthy that a positive association between topical tacrolimus and cutaneous lymphoma has been documented [33]. In another study, an 0.005% tacrolimus ophthalmic formulation was developed to treat patients with vernal keratoconjunctivitis. According to their study, even with the low tacrolimus concentration, symptom improvement was observed in patients with refractory VKC [34]. These results support the idea of the therapeutic power of tacrolimus in topical formulation and agree with our results as no recurrence or worsening of the ocular pathology was reported after the decrease of tacrolimus concentration in our formulation for any patient.

Regarding the VFQ-25 quality of life questionnaire, the patient’s perception of reduced pain may be attributed to the absence of the irritating effect of ethanol. Ethanol has a toxic effect on the corneal epithelium, although it has been used at higher concentrations (13%) in corneal surgeries such as keratectomy to remove the epithelium. Lower concentrations of ethanol are tolerable but are also accompanied by an irritant effect. In a previously published retrospective analysis of 20 mg/mL cyclosporine eye drops containing 25 mg/mL ethanol, 37% of treated patients reported burning sensations after application [19]. Also, the perception of reduced burning and itching sensations associated with data collected on the VAS scale was probably attributed to the removal of ethanol from the composition of the TCD eye drops.

The previous formulation of tacrolimus eye drops was poorly tolerated due to the presence of irritating excipients such as ethanol. To enhance tacrolimus solubility, cyclodextrins were presented as an effective and safe alternative [16]. In vitro studies have previously demonstrated the safety and good tolerability of the ophthalmic administration of cyclodextrins with poorly soluble drugs, such as corticosteroids or econazole [35]. The EMA has determined that 12.5% HPβCD solutions are not toxic or irritating to the eyes of rabbits [17]. García-Otero et al. analyzed the irritation of different cyclodextrins, including HPβCD, though Hen’s Egg Test on the Chorioallantois Membrane (HET-CAM) assay and Bovine Corneal Opacity and Permeability test (BCOP) [16]. In addition, ocular biopermanence was verified by in vivo Positron Emission Tomography (PET) [18]. Ex vivo, in vitro and in vivo assays concluded that formulations with HPβCD showed a well toxicity profile and enhanced biopermanence at the ocular level [16,18,19]. There has been an increase in the list of drugs containing cyclodextrins [36]. The main reasons for using these excipients are to reduce the instability of many molecules in aqueous media, e.g., hydrocortisone undergoing hydrolysis, and to reduce ocular irritation, such as pilocarpine which may precipitate and cause eye irritation and damage [37]. Within this context, there are currently 14 ophthalmic cyclodextrin formulations on the market, 8 of them containing HPβCDs [36].

To our knowledge, this is the first study to evaluate tear stability in patients under ophthalmic treatment with tacrolimus eye drops. Ethanol may modify tear stability as it functions as an organic solvent against the lipid layer of the tear [38]. It is known how tear osmolarity affects ocular comfort, with tear hyperosmolarity being one of the causes of discomfort and dry eye [39,40]. Comparing results, in our work it has been found that switching from TE from TCD treatment increased the TMH in at least half of the eyes evaluated. In contrast, the change in treatment produced no change in NIKBUT. All in all, it could be said that tear stability has remained stable, contributing to the effectiveness of the TCD treatment. Concerning the increased in OR after the first 3 months of TCD treatment, when an eye drop is administered, the interaction of the drop with the refractive surface and tear film may produce a slight irritability. In turn, viscosity above the limits may cause blurred vision, foreign body sensation and increased blinking rate, leading to an undesirable irritant effect of the ocular surface [41]. This disturbance would be directly increased by the density and viscosity of the eye drops and may cause some initial discomfort in patients with dryness symptoms or with long term treatments. Even with the objective data on OR, this event has not been correlated with other clinical and tear stability measurements.

The negative effects of steroids on IOP are known [42,43]. According to Miyazaki D. et al., tacrolimus treatment does not raise intraocular pressure although it may be influenced by concomitant use of corticosteroids or poor lid condition [44]. In this work, we studied the variation of intraocular pressure after switching from TE to TCD treatment in patients with concomitant treatments, some of them with corticosteroids. No variations in intraocular pressure were observed between visits, confirming that our 0.015% TCD eye drops do not raise intraocular pressure. These results are in agreement with other studies in which intraocular pressure has been evaluated in different presentations of tacrolimus for ophthalmic treatment [6,45].

In general, adherence to topical ocular treatments is quite low. One study analyzed subjective adherence to eye drops in inflammatory eye disease and concluded that 67% of patients were not adherent [46]. The main cause of lack of adherence is forgetfulness. Adherence questionnaires have significant limitations as subjectivity, memory bias (adherence can only be measured in a recent period), response bias (the patient answers what is expected of him) and the inability to detect unintentional adherence, that is, non-conscious forgetfulness [47]. Therefore, in this study, we attempted to measure adherence objectively by comparing the actual content used by the patient and the theoretical content according to the dosage regimen. Upon implementing this experimental method to calculate the compliance, we observed significant discrepancies in the weights, which could be attributed to a multitude of factors, including poor compliance and unintentional losses. The principal limitation of this analysis was the establishment of a cut-off point to differentiate between adherent and non-adherent patients, given that there is no existing literature on this subject. Finally, a margin of error of 35% was allowed between the actual and estimated weight, to consider accidental losses and evaporation losses. Patients classified as adherent according to the weighing system had reported 100% adherence on the VAS adherence scale.

To facilitate the transfer of this formulation to other centers, it is essential to emphasize that all the raw materials utilized are accessible to any HPD. In addition to this, no special equipment is required for its preparation, being all the apparatus used widely available in HPD.

## 4. Materials and Methods

### 4.1. Materials

Tacrolimus was acquired from Guinama^®^ S.L.U. (La Pobla de Vallbona, Spain), 2-hydroxypropyl-β-cyclodextrin Kleptose^®^ HPB (HPβCD; MW = 1399 Da, substitution degree = 0.65 molar ratio) was provided from Roquette Laisa S.A.^®^ (Valencia, Spain), Liquifilm^®^ was purchased from Allergan^®^ Pharmaceuticals Ireland (Mayo, Ireland), Balanced Salt Solution (BSS^®^) was acquired from Alcon^®^ laboratories (FortWorth, TX, USA) and Prograf^®^ (5 mg/mL, ampoules) was purchased from Astellas Pharma S.A.^®^ (Madrid, Spain).

### 4.2. Elaboration and Packaging of Tacrolimus Sterile Solutions

Two different tacrolimus formulations were prepared, a 0.03% tacrolimus formulation using intravenous drug presentation (Prograf^®^) (TE) and a 0.015% tacrolimus formulation (without ethanol) with 40% HPβCD (TCD). Both compounded formulations were elaborated in sterile conditions under a vertical flow cabinet. TE was elaborated following Luaces-Rodriguez et al. previous work [11]. The necessary volume of tacrolimus (Prograf^®^ 5 mg/mL) was added to a luer-lock syringe to reach a concentration of 0.03%. The vehicle used for its preparation was Liquifilm^®^ (LI), an artificial tear based on polyvinyl alcohol 1.4% *w*/*v*. Afterward, the solution was filtered through a 5 µm filter (RoweMed AG—Medical for life, Parchim, Germany) and filled into 5 mL High-Density Polyethylene (HDPE) eye drop containers (Envases Farmacéuticos Sirep, S.L., Tarragona, Spain).

To elaborate TCD compounded formulation, HPβCD was added over 2/3 of the final volume of LI to reach a concentration of 40% under intense magnetic stirring. Later, it was left under agitation at low stirring speed for a total of 24 h to eliminate foam. Tacrolimus was added under intense stirring to obtain a concentration of 0.015%, heating the solution to 40 °C during the addition. The solution was left in agitation for a total of 96 h to achieve maximum tacrolimus solubilization. Finally, it was made up to the final volume with LI and filtered with a 0.22 µm membrane filter (vacuum-driven bottles FPR204250 PES 0.22 µm; Biofil, Alicante, Spain) under vacuum. Both formulations were finally packaged in 5 mL HDPE eye drops containers.

### 4.3. Study Design and Visits

An open-label, sequential, prospective, single-center, 3-month study was conducted at the Ophthalmology Department of the University Clinical Hospital of Santiago de Compostela. The present study complied with the tenets of the Declaration of Helsinki and was approved by the Institutional Review Board/Ethics Committee of the Ethical Committee of Clinical Research of Galicia (AFF-FOR-2019-01). For inclusion, patients had to have been under treatment with the TE formulation for, at last, 6 months and agree to participate by signing the informed consent form. The only exclusion criteria were that the patient refused to participate or did not sign the informed consent form.

**Figure 4:**
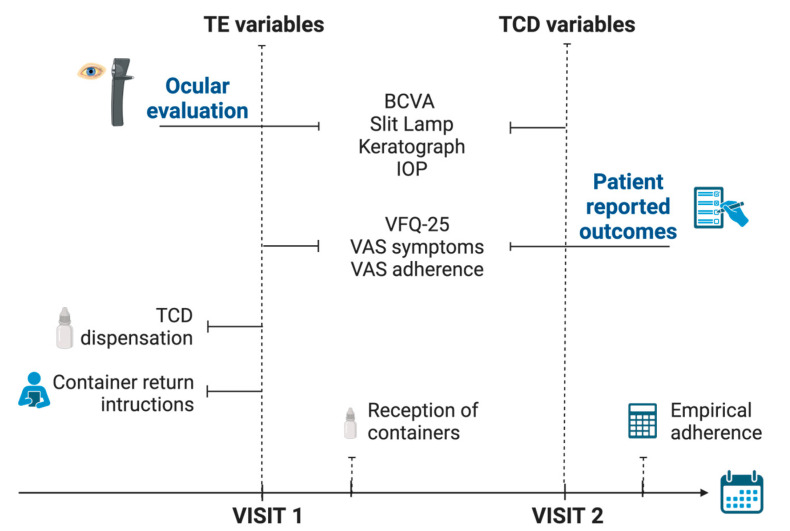
Study design: visits, collected variables and administration of the study medication. BCVA: best-corrected visual acuity; IOP: Intraocular pressure; VFQ-25: Visual Function Questionnaire; TCD: tacrolimus 0.015% in cyclodextrin; TE: tacrolimus 0.03% prepared from commercial intravenous presentation Prograf^®^; VAS: visual analog scale. Created in BioRender.

Two visits were planned for the study. At visit 1 (baseline visit), variables related to TE formulations were collected and the new TCD formulation was dispensed. In the second visit, that took place three months after TCD were dispensed, variables regarding TCD eye drops were collected. Study design and visits are shown in Figure 4.

### 4.4. Data Collection and Clinical Evaluation

Demographic (age, gender), clinical (diagnosis, affected eye) and treatment characteristics (dosage regimen, duration, adverse effects, concomitant treatment) were collected through review of medical records and interviews with the patient. To evaluate the effectiveness and safety of TE and TCD eye drops, a clinical examination was performed at each visit, from less invasive tests to more invasive tests to avoid interference. To avoid the influence of diurnal variations in tear and intraocular pressure parameters, all measurements were performed within the same time range for both visits (8:30 a.m.–14:00 p.m.). Clinical evaluation and intraocular pressure were performed only by two ophthalmologists, who in turn employed scales commonly used in this type of studies, in an attempt to reduce subjectivity as much as possible (Tables S1 and S2 of the Supplementary Material). The rest of the clinical tests were carried out by a single optometrist. To avoid bias, data collection and data analyzed was performed by different researchers.

#### Clinical Evaluation

The ocular evaluation began with the measurement of the BCVA using a Snellen test. If the patient was wearing correction at that moment, the measurement was made with the patient’s own spectacles. BCVA improvement was considered when the visual acuity at visit 2 was greater than visual acuity at visit 1, i.e., when patients discerned at least one more optotype line relative to the initial visit. Those patients who only perceived light, with or without projection were excluded, and values were transform in LogMar notation for the correct comparison and analysis [48]. Ocular signs were examined with a slit lamp (Topcon Europe Medical B.V., Barcelona, Spain), consisting of a study of the conjunctiva, presence of papillae, presence of trantas dots on the limbus and presence of corneal signs. For each item, a 4-point classification was performed as shown in Table S1 of the Supplementary Material. For these parameters, a decrease in one grade scale in visit 2 in comparison with visit 1 was considering to be an improvement in the clinical sign evaluated. To assess the corneal fluorescein staining a slit lamp was used and classified using the Oxford corneal staining scheme [49] (Table S2, Supplementary Material). For this analysis, a decrease in Oxford scale between visits was associated with a decrease in corneal staining.

Lacrimal stability and OR were studied using the OCULUS Keratograph 5M^®^ (Oculus, Wetzlar, Germany) and Oculus TF-Scan module (Oculus, Wetzlar, Germany). In the study of lacrimal stability and OR, those eyes whose pathology prevented the proper functioning of the optics of the instruments were excluded. According to our previous work [50], lacrimal stability was measured though the TMH, and the NIKBUT. Both parameters were measured in triplicate. The degree of OR provided by the instrument of the TB and NB area and a TL and NL conjunctiva was measured, as well as the average degree of ocular redness. For TMH and NIKBUT analysis, higher values in visit 2 in comparison with the initial visit were considered an improvement, while improvement in OR was considered if its value decreased over the course of the visits.

With the purpose of verifying that the switch of tacrolimus formulation did not affect the IOP, this was assessed at both visits using Perkins’s tonometer (Perkins MK2, Haag-Streig Holding, Harlow, UK). IOP measurements were carried out as the last clinical test to avoid the possible influence in the other parameters since previous administration of a topical anesthetic (Colircusí Fluotest^®^, Alcon Healthcare, TX, USA) was needed. To minimize instrument variability, the tonometer was calibrated before each visit and the measurement was carried out with the patient in a sitting position. In the analysis of the clinical ocular parameters, those patients who had no measurements at both visits were excluded.

### 4.5. Patient Reported Outcomes and Compliance

#### 4.5.1. Patient Reported Outcomes

In both initial and second visits patients completed the VFQ-25 (Table S3, Supplementary Material) developed by the National Eye Institute [51], which provides information about items grouped into 12 domains: near vision, difficulty distance vision, social functioning, role limitations due to vision, dependency on others due to vision, mental health symptoms due to vision, driving difficulties, peripheral vision, color vision and ocular pain. Additionally, the VFQ-25 contains a single general health rating question and a single question about global vision. The questionnaire was completed by all patients aged 11 years or older, and those patients who could not complete it themselves received help from companions and research team. The VFQ scale score was established considering that each item has a score range from 0 to 100. A high score indicates excellent functionality and well-being. The overall score was defined by establishing the average per domain and subsequently the scores obtained in each domain were averaged.

Response to treatment perceived by the patient was evaluated using a VAS of symptoms (burning, discharge, tearing, foreign body sensation, pain, photophobia) (Table S4, Supplementary Material) performed at the initial visit (when treated with TE) and at the follow-up visit (when treated with TCD). Patients rated the intensity of these symptoms from 0 to 10 (highest degree of intensity) [52].

#### 4.5.2. Compliance

The level of compliance to treatment was performed at the initial visit (regarding to TE) and three months after the beginning of TCD. Subjective adherence was evaluated through a VAS of adherence questionnaire (Table S5, Supplementary Material) scored from 0 (not adherent) to 10 (fully adherent) [53].

Objective adherence to TCD eye drops was determined by the difference in weight between the bottles before and after use. Patients were instructed at visit 1 to store two containers of used TCD eye drops to return them to the HPD for adherence analysis. Before dispensing to the patient and after use, the two containers of TCD were weighed. In parallel, the theoretical amount used by the patient was calculated based on the dosing regimen and the average weight per drop (0.0444 mg/drop). Finally, the difference between theoretical and actual use was calculated. A margin of error of 35% was assumed to account for the possibility of accidental loss of eye drops.

### 4.6. Statistical Analysis

The results of the different assays were plotted using Graph Pad Prism^®^ v.9.0.1 software (GraphPad Software, San Diego, CA, USA) and IBM Corp. Released 2021. IBM SPSS Statistics for Windows, Version 28.0. Armonk, NY, USA: IBM Corp. To evaluate the normality of the data, the Kolmogorov Smirnov test was used. To carry out the significance analysis of the data obtained, the Wilcoxon test was performed. Spearman rank correlation was also used to characterize the relationship between two variables. *p* values < 0.05 were considered statistically significant.

## 5. Conclusions

The use of cyclodextrins as an excipient of ocular pharmaceutical compounds is a promising alternative to solubilize lipophilic active principles and eliminate irritating excipients, such as those used in the preparation of the ophthalmic tacrolimus compounded formulation. The administration of TCD eye drops for a period of three months resulted in the maintenance of stable ocular clinical signs, tear stability and intraocular pressure. Furthermore, the patient-reported damage was also favorable, particularly in terms of pain and burning. Decreasing the concentration of tacrolimus in our formulation may be a appropriate alternative to reduce the dose of immunosuppression, allowing to mitigate the mutagenic risk of the drug in long-term use. Further studies with larger populations and longer study periods are needed to confirm these findings.

## Figures and Tables

**Table 1:** Analysis of clinical parameters evaluated for the effectiveness of the new tacrolimus formulation and tear stability parameters (TMH and NIKBUT). Percentage of evaluated eyes maintaining equal, better, or worse values at visit 2 (TCD) compared to visit 1 (TE).

	n (Eyes)	Equal (%)	Improvement (%)	Worsened (%)
BCVA	69	27.54	44.93	27.54
Ocular signs				
Conjunctiva	76	56.58	17.11	26.32
Papillae	76	63.16	9.21	27.63
Tantras points	76	76	-	-
Corneal sign	76	81.58	18.42	0.0
Oxford	76	72.37	25.0	2.63
TMH	73	2.74	54.79	42.47
NIKBUT	65	3.08	44.62	52.31

BCVA: best corrected visual acuity; n: population size; NIKBUT: noninvasive keratograph break up time; TMH: tear meniscus height.

**Table 2:** Median (Md) and percentiles (Q_1_–Q_3_) of tear stability parameters (TMH and NIKBUT), ocular redness (OR) and intraocular pressure (IOP) and the comparison between visits.

			TE	TCD	*p*-Value
		n (Eyes)	Md (Q_1_–Q_3_)	Md (Q_1_–Q_3_)	
TMH (mm)		73	0.25 (0.19–0.20)	0.26 (0.36–0.39)	0.2404
NIKBUT (s)		65	8.79 (6.26–5.38)	7.66 (15.01–12.70)	0.0922
OR	TB	63	1.0 (0.6–1.6)	1.2 (0.7–1.8)	0.0168
	NB	63	1.2 (0.8–2.1)	1.4 (0.9–2.0)	0.2199
	TL	63	0.5 (0.4–1.1)	0.8 (0.5–1.2)	0.0294
	NL	63	0.6 (0.4–1.4)	0.8 (0.5–1.4)	0.0182
	ORT	63	1.1 (0.7–1.8)	1.3 (0.8–1.8)	0.0829
	AREA (mm^3^)	63	10.95 (7.4–13.83)	9.90 (6.9–15.08)	0.8489
IOP (mmHg)		56	14.0 (12.0–16.0)	14.0 (12.0–15.75)	0.7589

AREA: total area analyzed in mm^3^; IOP: intraocular pressure; Md: median; n: population size; NB: nasal bulbar area; NIKBUT: noninvasive keratograph break up time; NL: nasal limbal; Q_1_: 25th percentile; Q_3_: 75th percentile; TB: temporal bulbar area; TCD: tacrolimus 0.015% in cyclodextrin; TE: tacrolimus 0.03% prepared from commercial intravenous presentation Prograf^®^; TL: temporal limbal; TMH: tear meniscus height; ORT: average ocular redness.

**Table 3:** Percentage of patients who improved or worsened in both eyes or in at least one of them, and those who remained with the same symptoms on the VAS of symptoms. The inconclusive section includes those patients who, with both eyes affected, improved in one eye and worsened in the other.

	Burning	Discharge	Lacrimation	FBS	Pain	Photophobia
n	33	35	36	35	33	33
Improved ≥ 2 points	48.5%	22.9%	27.8%	40%	27.3%	27.3%
Improved 1 point	3.1%	11.4%	11.1%	5.7%	6.1%	12.1%
Deterioration ≥ 2 points	15.2%	17.1%	5.6%	17.1%	12.1%	21.2%
Deterioration 1 point	3.1%	5.7%	11.1%	8.6%	9.1%	12.1%
Same symptoms	24.3%	40%	38.8%	25,7%	42.4%	27.3%
Inconclusive	6.1%	2.9%	5.6%	2,9%	3%	-

FBS: foreign body sensation; n: population size.

## Data Availability

Data are contained within the article and Supplementary Materials.

## Supplementary Materials

The following supporting information can be downloaded at: https://www.mdpi.com/article/10.3390/ijms25189847/s1.
