# Supplementary material for: Clinical Effectiveness, Safety, and Compliance of Two Compounded Formulations of Tacrolimus Eye Drops: An Open-Label, Sequential Prospective Study
Source: Int J Mol Sci. 2024 Sep 12;25(18):9847. doi: 10.3390/ijms25189847 (PMC11432331; doi:10.3390/ijms25189847)
Supplement: Supplementary file 1 [file ijms-25-09847-s001.zip › ijms-3165366-supplementary.pdf]

Clinical Effectiveness, Safety, and Compliance of Two Compounded Formulations of Tacrolimus Eye Drops: An Open-Label, Sequence Prospective Study

María Puente-Iglesias [1,2,3†], Andrea Cuartero-Martínez [1,2,3†], Rosario Touriño-Peralba [4], María Teresa Rodríguez-Ares [4], María Jesús Giráldez [5], Eva Yebra-Pimentel [5], Laura García-Quintanilla [1,2], Xurxo García-Otero [3], Miguel González-Barcia [1,2], Irene Zarra Ferro [1,2], Francisco J Otero-Espinar [3], Anxo Fernández-Ferreiro [1,2*] and Ana Castro-Balado[1,2*]

Table S1. Four-point classification of the ocular clinical signs evaluated. The numerical classification follows an ascending direction according to the severity of the sign, where 0 represents the absence of the evaluated sign. OD: Oculus Dexter, right eye. OS: Oculus Sinister, left eye.

**OD**					
**Conjunctiva**	3	Important vessel dilatation	**Trantas dots on the limbus**	3	>9 dots
	2	Moderate vessel dilatation		2	8-9 dots
	1	Mild vessel dilatation		1	1-4 dots
	0	No dilatation		0	No dots
**Papillae**	3	Giant (>1 mm and elevated)	**Corneal signs**	3	Corneal ulcer or erosion
	2	Moderate (0.5-0.9 mm and flat)		2	Superficial punctate keratitis exfoliation
	1	Mild (0.1-0.4 mm and flat)		1	Superficial punctate keratitis
	0	Normal		0	Normal
**OS**					
**Conjunctiva**	3	Important vessel dilatation	**Trantas dots on the limbus**	3	>9 dots
	2	Moderate vessel dilatation		2	8-9 dots
	1	Mild vessel dilatation		1	1-4 dots
	0	No dilatation		0	No dots
**Papillae**	3	Giant (>1 mm and elevated)	**Corneal signs**	3	Corneal ulcer or erosion
	2	Moderate (0.5-0.9 mm and flat)		2	Superficial punctate keratitis exfoliation
	1	Mild (0.1-0.4 mm and flat)		1	Superficial punctate keratitis
	0	Normal		0	Normal

OD: Oculus Dexter, right eye. OS: Oculus Sinister, left eye.

Table S2. Illustration adapted from the Oxford fluorescein corneal staining scheme. Examiners should perform a visual examination of the corneal staining present and compare it with the figure that most closely resembles the one provided.

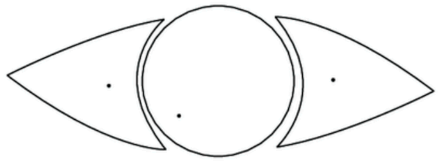	Figure A<br>Equal to or less than in figure B<br>Grade 0
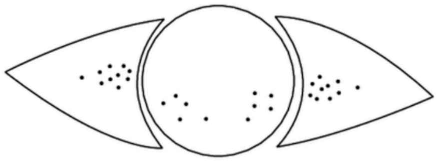	Figure B<br>More than figure A and equal to or less than figure B<br>Grade 1
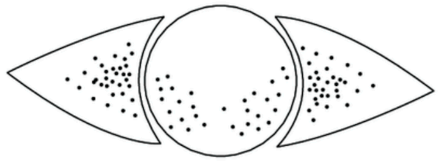	Figure C<br>More than figure B and equal to or less than figure C<br>Grade 2
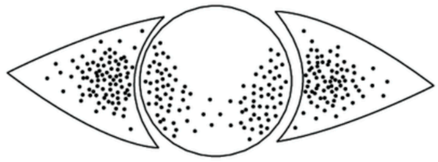	Figure D<br>More than figure C and equal to or less than figure D<br>Grade 3
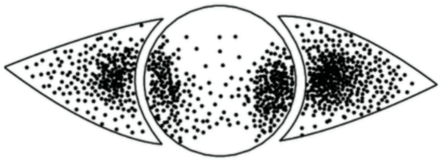	Figure E<br>More than figure D and equal to or les than figure E<br>Grade 4
	More than figure E<br>Grade 5

Table S3. National Eye Institute Visual Functioning Questionnaire – 25 (VFQ-25)

PART 1 - GENERAL HEALTH AND VISION (Circle One):	
**1. In general, would you say your overall health is:**	
Excellent	100
Very good	75
Good	50
Fair	25
Poor	0
**2. At the present time, would you say your eyesight using both eyes (with glasses or contact lenses, if you wear them) is excellent, good, fair, poor, ¿or very poor or are you completely blind?**	
Excellent	100
Very good	80
Fair	60
Poor	40
Very poor	20
Completely Blind	0
**3. How much of the time do you worry about your eyesight?**	
None of the time	100
A little of the time	75
Some of the time	50
Most of the time	25
All of the time	0
**4. How much pain or discomfort have you had in and around your eyes (for example, burning, itching, or aching)? Would you say it is:**	
None	100
Mild	75
Moderate	50
Severe	25
Very severe	0
PART 2 - DIFFICULTY WITH ACTIVITIES<br>The next questions are about how much difficulty, if any, you have doing certain activities wearing your glasses or contact lenses if you use them for that activity.	
**5. How much difficulty do you have reading ordinary print in newspapers? Would you say you have:**	
No difficulty at all	100
A little difficulty	75
Moderate difficulty	50
Extreme difficulty	25
Stopped doing this because of your eyesight	0
Stopped doing this for other reasons or not interested in doing this	**

6. How much difficulty do you have doing work or hobbies that require you to see well up close, such as cooking, sewing, fixing things around the house, or using hand tools? Would you say:

No difficulty at all	100
A little difficulty	75
Moderate difficulty	50
Extreme difficulty	25
Stopped doing this because of your eyesight	0
Stopped doing this for other reasons or not interested in doing this	**

7. Because of your eyesight, how much difficulty do you have finding something on a crowded shelf?

No difficulty at all	100
A little difficulty	75
Moderate difficulty	50
Extreme difficulty	25
Stopped doing this because of your eyesight	0
Stopped doing this for other reasons or not interested in doing this	**

8. How much difficulty do you have reading street signs or the names of stores?

No difficulty at all	100
A little difficulty	75
Moderate difficulty	50
Extreme difficulty	25
Stopped doing this because of your eyesight	0
Stopped doing this for other reasons or not interested in doing this	**

9. Because of your eyesight, how much difficulty do you have going down steps, stairs, or curbs in dim light or at night?

No difficulty at all	100
A little difficulty	75
Moderate difficulty	50
Extreme difficulty	25
Stopped doing this because of your eyesight	0
Stopped doing this for other reasons or not interested in doing this	**

10. Because of your eyesight, how much difficulty do you have noticing objects off to the side while you are walking along?

No difficulty at all	100
A little difficulty	75
Moderate difficulty	50
Extreme difficulty	25
Stopped doing this because of your eyesight	0
Stopped doing this for other reasons or not interested in doing this	**

11. Because of your eyesight, how much difficulty do you have seeing how people react to things you say?

No difficulty at all	100
A little difficulty	75
Moderate difficulty	50
Extreme difficulty	25
Stopped doing this because of your eyesight	0
Stopped doing this for other reasons or not interested in doing this	**

12. Because of your eyesight, how much difficulty do you have picking out and matching your own clothes?

No difficulty at all	100
A little difficulty	75
Moderate difficulty	50
Extreme difficulty	25
Stopped doing this because of your eyesight	0
Stopped doing this for other reasons or not interested in doing this	**

13. Because of your eyesight, how much difficulty do you have visiting with people in their homes, at parties, or in restaurants?

No difficulty at all	100
A little difficulty	75
Moderate difficulty	50
Extreme difficulty	25
Stopped doing this because of your eyesight	0
Stopped doing this for other reasons or not interested in doing this	**

14. Because of your eyesight, how much difficulty do you have going out to see movies, plays, or sports events?

No difficulty at all	100
A little difficulty	75
Moderate difficulty	50
Extreme difficulty	25
Stopped doing this because of your eyesight	0
Stopped doing this for other reasons or not interested in doing this	**

15. Now, I'd like to ask about driving a car. ¿Are you currently driving, at least once in a while?

Yes → Skip to question 15c

No → Skip to question 15a

15a. IF NO, ASK: ¿Have you never driven a car or have you given up driving?

Never drop → Skip to question 17

Gave up → Skip to question 15b

15b. IF GAVE UP DRIVING: Was that mainly because of your eyesight, mainly for some other reason, ¿or because of both your eyesight and other reasons?

Mainly eyesight → Skip to question 17

Mainly other reasons → Skip to question 17	
Both eyesight and other reasons → Skip to question 17	

15c. IF CURRENTLY DRIVING: ¿How much difficulty do you have driving during the daytime in familiar places? Would you say you have:

No difficulty at all	100
A little difficulty	75
Moderate difficulty	50
Extreme difficulty	25

16. How much difficulty do you have driving at night? Would you say you have:

No difficulty at all	100
A little difficulty	75
Moderate difficulty	50
Extreme difficulty	25
Stopped doing this because of your eyesight	0
Stopped doing this for other reasons or are you not interested	**

16a. How much difficulty do you have driving in difficult conditions, such as in bad weather, during rush hour, on the freeway, or in city traffic? Would you say you have:

No difficulty at all	100
A little difficulty	75
Moderate difficulty	50
Extreme difficulty	25
Stopped doing this because of your eyesight	0
Stopped doing this for other reasons or are you not interested	**

ART 3: RESPONSES TO VISION PROBLEMS

The next questions are about how things you do may be affected by your vision. For each one, I'd like you to tell me if this is true for you all, most, some, a little, or none of the time.

17. Do you accomplish less than you would like because of your vision?

All of the time	0
Most of the time	25
Some of the time	50
A Little of the time	75
None of the time	100

18. Are you limited in how long you can work or do other activities because of your vision?

All of the time	0
Most of the time	25
Some of the time	50
A Little of the time	75
None of the time	100

19. How much does pain or discomfort in or around your eyes, for example, burning, itching, or
aching, keep you from doing what you'd like to be doing? Would you say:

All of the time	0
Most of the time	25
Some of the time	50
A Little of the time	75
None of the time	100

20. I stay home most of the time because of my eyesight:

Definitely true	0
Mostly true	25
Not sure	50
Mostly false	75
Definitely false	100

21. I feel frustrated a lot of the time because of my eyesight:

Definitely true	0
Mostly true	25
Not sure	50
Mostly false	75
Definitely false	100

22. I have much less control over what I do, because of my eyesight:

Definitely true	0
Mostly true	25
Not sure	50
Mostly false	75
Definitely false	100

23. Because of my eyesight, I have to rely too much on what other people tell me:

Definitely true	0
Mostly true	25
Not sure	50
Mostly false	75
Definitely false	100

24. I need a lot of help from others because of my eyesight:

Definitely true	0
Mostly true	25
Not sure	50
Mostly false	75
Definitely false	100

25. I worry about doing thing that will embarrass myself or others, because of my eyesight:

Definitely true	0
Mostly true	25
Not sure	50

	Mostly false	75
	Definitely false	100

Table S4. Visual Analogue Scale - Questionnaire concerning symptoms of eye discomfort. Patients were provided with an individual test for each eye to be examined. A score of 0-3 indicates mild intensity, 4-8 moderate intensity and 9-10 intense intensity.

**Burning (1)**	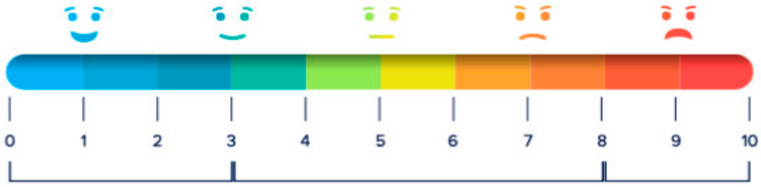
**Discharge (2)**	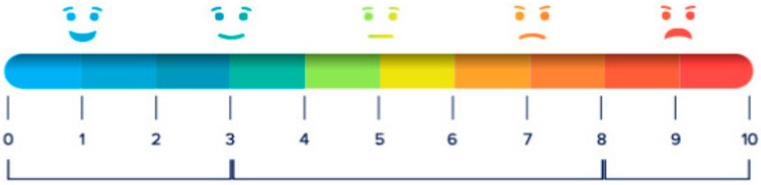
**Tearing (3)**	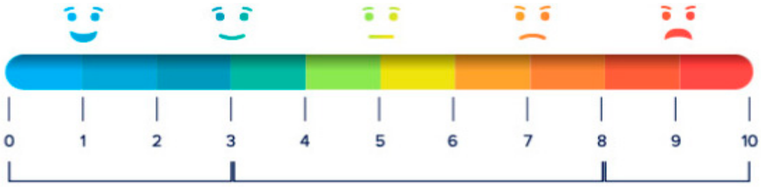
**Foreign body sensation (4)**	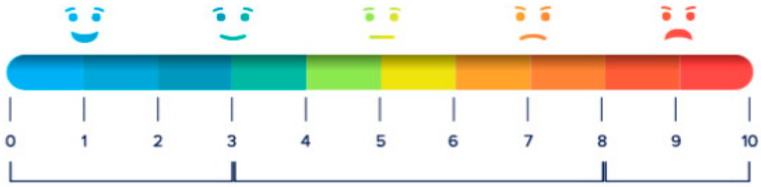
**Pain (5)**	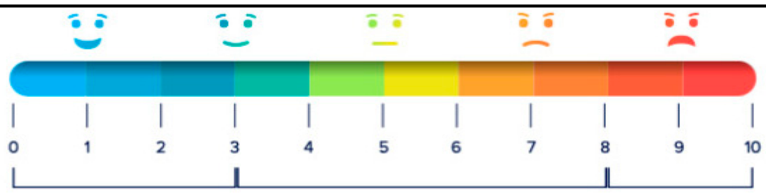
**Photophobia (6)**	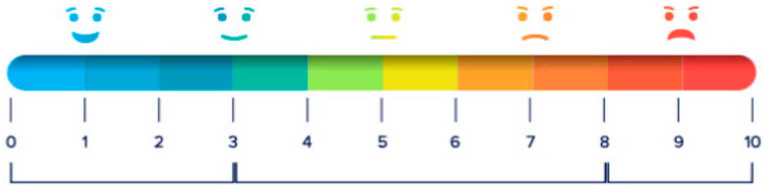

Table S5. Representation of the Visual Analogue Scale provided to patients concerned to therapeutic adherence and the instructions to complete the questionnaire.

How did you take the medicine you pick up at the hospital? (To be filled in by the patient with a black pen)

Please mark on the line below the item that best defines HOW you have taken the medication DURING THE LAST 30 DAYS:

- 0% means you have not taken ANY doses of medication.
- 50% if you have taken HALF of the prescribed dosages
- 100% if you have taken ALL your medications at the prescribed times and without missing any day.

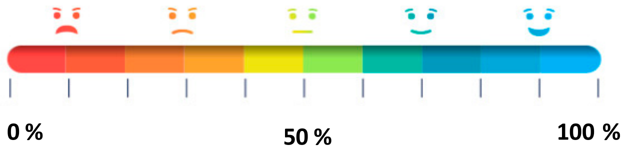

Table S6. Median and percentiles of the three measurements taken at both visits for each of the patients included in the study for the tear meniscus height measurement (THM).

	TE			TCD		
	**Md**	**Q1**	**Q3**	**Md**	**Q1**	**Q3**
**OD-001**	0,26	0,26	0,27	0,28	0,25	0,31
**OS-001**	0,24	0,24	0,26	0,32	0,31	0,32
**OD-003**	0,42	0,39	0,46	0,15	0,13	0,35
**OS-003**	0,24	0,23	0,32	0,25	0,22	0,27
**OD-006**	0,22	0,22	0,23	0,4	0,29	0,55
**OD-007**	0,57	0,55	0,59	0,77	0,65	0,78
**OS-007**	0,58	0,58	0,58	0,33	0,32	0,39
**OS-008**	0,28	0,27	0,28	0,24	0,23	0,24
**OS-009**	0,38	0,29	0,43	0,32	0,28	0,37
**OD-011**	0,22	0,22	0,23	0,27	0,24	0,28
**OS-011**	0,24	0,21	0,25	0,36	0,36	0,38
**OD-012**	0,29	0,26	0,32	0,19	0,18	0,22
**OS-012**	0,19	0,15	0,21	0,18	0,17	0,19
**OD-013**	0,22	0,18	0,22	0,21	0,19	0,23
**OS-013**	0,24	0,2	0,28	0,33	0,28	0,36
**OD-014**	0,22	0,17	0,22	0,24	0,2	0,24
**OD-017**	0,64	0,62	1,19	0,42	0,32	0,49
**OS-017**	0,58	0,56	0,6	0,76	0,42	0,96
**OD-018**	0,18	0,12	0,22	0,22	0,19	0,23
**OD-019**	0,2	0,19	0,22	0,37	0,36	0,38
**OD-020**	0,17	0,15	0,19	0,18	0,17	0,2
**OD-021**	0,19	0,17	0,21	0,27	0,27	0,27

**OD-022**	0,27	0,23	0,29	0,41	0,35	0,42
**OS-022**	0,45	0,41	0,49	0,42	0,42	0,58
**OD-023**	0,24	0,23	0,39	0,23	0,21	0,3
**OS-023**	0,17	0,14	0,18	0,2	0,17	0,21
**OD-026**	0,33	0,32	0,33	0,32	0,31	0,33
**OS-026**	0,33	0,29	0,34	0,41	0,39	0,42
**OD-027**	0,25	0,18	0,27	0,26	0,22	0,31
**OS-027**	0,24	0,24	0,26	0,22	0,11	0,24
**OS-028**	0,13	0,11	0,15	0,15	0,13	0,15
**OD-028**	0,13	0,13	0,14	0,17	0,17	0,18
**OD-029**	0,22	0,22	0,24	0,19	0,15	0,27
**OS-029**	0,24	0,24	0,27	0,2	0,18	0,23
**OD-030**	0,34	0,28	0,36	0,17	0,16	0,18
**OS-030**	0,17	0,17	0,2	0,17	0,12	0,22
**OD-031**	0,37	0,36	0,45	0,36	0,33	0,36
**OS-031**	0,81	0,76	0,85	0,325	0,28	0,37
**OD-032**	0,49	0,43	0,56	0,44	0,41	0,46
**OS-032**	0,82	0,77	0,84	0,64	0,61	0,66
**OD-034**	0,96	0,71	1,21	0,19	0,13	0,23
**OS-034**	0,13	0,11	0,18	0,1	0,1	0,17
**OD-035**	0,14	0,11	0,14	0,27	0,25	0,27
**OS-035**	0,19	0,13	0,22	0,22	0,18	0,23
**OD-036**	0,96	0,96	1,04	0,83	0,73	0,83
**OS-037**	0,13	0,08	0,13	0,13	0,13	0,17
**OD-038**	0,17	0,13	0,17	0,19	0,15	0,19
**OS-038**	0,22	0,2	0,23	0,14	0,13	0,18
**OD-039**	0,99	0,98	1	1,22	1,02	1,45
**OS-039**	0,69	0,65	0,7	1,12	0,65	1,67
**OD-040**	0,2	0,18	0,2	0,23	0,15	0,25
**OS-040**	0,31	0,31	0,36	0,38	0,33	0,41
**OS-041**	0,2	0,2	0,27	0,305	0,29	0,32
**OD-042**	0,55	0,48	0,7	0,42	0,4	0,57
**OS-042**	0,53	0,48	0,62	0,74	0,6	0,83
**OD-043**	0,17	0,17	0,19	0,19	0,17	0,2
**OS-043**	0,15	0,14	0,15	0,24	0,23	0,27
**OD-044**	0,24	0,23	0,28	0,36	0,31	0,38
**OS-044**	0,18	0,15	0,22	0,41	0,34	0,41
**OD-045**	0,15	0,13	0,15	0,2	0,19	0,23
**OS-045**	0,17	0,14	0,17	0,13	0,1	0,15
**OD-046**	0,3	0,3	0,35	0,33	0,29	0,36
**OS-046**	0,37	0,31	0,37	0,28	0,27	0,32
**OD-049**	0,2	0,17	0,24	0,13	0,09	0,14
**OS-049**	0,2	0,17	0,2	0,19	0,17	0,21
**OS-050**	0,55	0,53	0,55	0,29	0,28	0,33
**OD-051**	0,25	0,23	0,31	0,24	0,23	0,29
**OS-051**	0,24	0,23	0,31	0,22	0,22	0,29

**OD-052**	0,15	0,13	0,17	0,18	0,13	0,18
**OD-054**	0,34	0,31	0,38	0,46	0,44	0,47
**OS-054**	0,27	0,24	0,38	0,41	0,37	0,42
**OD-056**	0,2	0,17	0,21	0,92	0,73	0,96
**OS-056**	0,22	0,22	0,23	0,38	0,38	0,43

Md: median; OD: oculus dexter, right eye; OI: oculus sinister, left eye; Q1: 25th percentile; Q3: 75th percentile; TCD: tacrolimus 0.015% in cyclodextrin; TE: tacrolimus 0.03% prepared from commercial intravenous presentation Prograf®.

Table S7. Median and percentiles of the three measurements taken at both visits for each of the patients included in the study of noninvasive keratograph break up time.

	**TE**			**TCD**		
	**Md**	**Q1**	**Q3**	**Md**	**Q1**	**Q3**
**OD-001**	10	10	14,53	7	5,99	10
**OS-001**	10,83	7	11,85	5,74	4,59	10
**OD-003**	5,67	4,65	7,26	5,61	5,23	8,67
**OS-003**	3	2	3,95	6,82	6,44	7,52
**OS-009**	3,565	3,12	4,01	2,87	2,87	2,87
**OD-011**	11,28	9,5	24	13,77	9,11	23,45
**OS-011**	21,16	14,02	24	7,84	5,1	13,38
**OD-012**	7,78	2,36	24	3	2,36	11
**OS-012**	7,33	5,61	7,71	2	2	5,23
**OD-013**	6,31	2,61	9,62	2,61	1	7,52
**OS-013**	4,78	3,06	5,22	5,35	2,87	10
**OD-014**	7,14	4,52	11,28	7,33	4,01	10,9
**OD-017**	7,78	1	11	8,28	8,16	16,76
**OS-017**	22,43	16,7	24	20,01	11,28	23,9
**OD-019**	2	2	2	3,5	2	5,74
**OD-020**	4,33	4,33	4,33	5,67	4,21	10,13
**OD-021**	8	3,5	9	4,14	2,42	4,91
**OD-022**	4,59	4,46	18,35	10,64	4,21	14,53
**OS-022**	6,69	5,74	6,95	8,09	6,12	24
**OD-023**	3,25	2,17	24	7,52	4,21	8,54
**OS-023**	7,46	2,42	9	2	1,34	9,5
**OD-026**	24	24	24	24	12,11	24
**OS-026**	11,66	5,42	22,05	6,44	3,12	9,62
**OD-027**	6,37	2,87	11,47	8	4,4	24
**OS-027**	7,77	2	13	13,83	11,34	24
**OD-028**	14,72	6,56	24	11,09	8	22,62
**OS-028**	24	5,03	24	9,49	3,89	9,81
**OD-029**	7,97	4,46	10	4,59	1,98	4,84
**OS-029**	4,91	3,63	6,44	13	3,7	23,07
**OS-030**	6,31	4	6,5	7,14	3,5	8,03
**OD-031**	24	8,03	24	17,65	7,58	23,2
**OS-031**	21,86	13,57	24	6,5	5,23	24
**OD-032**	8,79	7,52	9,75	5,03	3,5	11,15

**OS-032**	4,33	2,93	17,84	14,72	7,2	24
**OD-034**	2,42	2,36	2,48	6,12	3,06	6,75
**OS-034**	4,27	2	4,78	2	1	6,12
**OD-035**	5,42	4,78	21,67	15,29	6,25	16,44
**OS-035**	12,49	8,22	19,82	3,19	2	6,05
**OD-036**	13,77	11,66	22,33	24	13,57	24
**OD-038**	19,5	19,25	24	9,37	3,25	11
**OS-038**	10,96	3,95	24	16,38	13,32	22,62
**OD-039**	6,12	4,59	24	8,03	7	15,99
**OS-039**	6	5	9,05	3	3	4,91
**OS-040**	5	3,7	10	15,49	2,42	23,77
**OS-041**	8,86	4,78	12,74	4,27	3	5,74
**OD-042**	18,1	4,14	21	20,97	16,95	23,01
**OS-042**	7,46	3,06	18,23	23,01	21,09	24
**OD-043**	24	15,87	24	24	12,74	24
**OS-043**	21,73	5	24	6,76	5,54	13,89
**OD-044**	24	24	24	11,53	7,84	12,87
**OS-044**	24	4,27	24	13,83	8	18,23
**OD-045**	4,78	4,33	7,71	4,97	4,72	24
**OS-045**	10,77	4,27	11,28	4,91	1	19,95
**OD-046**	2,17	2	2,17	2,68	2	2,74
**OS-046**	3	3	3,06	2,23	1,85	3,5
**OD-049**	11,79	3,06	12,55	10,58	9,94	13,45
**OS-049**	7,9	6,56	8,41	8	3,44	8,92
**OS-050**	2	1	24	7,33	5,61	11,66
**OD-051**	23,83	3,76	24	5,48	2	13,45
**OS-051**	10,71	7,65	24	7,33	2,68	12,23
**OD-052**	24	3,95	24	3,76	3,19	7,78
**OD-054**	4,91	4,08	10,77	4,4	3,76	11,47
**OS-054**	3,89	3,89	12,24	5,42	5,04	8,03
**OD-056**	3,31	1	12,87	4,14	1	8,22
**OS-056**	12	10,26	24	6,18	6,18	24

Md: median; OD: oculus dexter, right eye; OS: oculus sinister, left eye; Q1: 25th percentile; Q3: 75th percentile; TCD: tacrolimus 0.015% in cyclodextrin; TE: tacrolimus 0.03% prepared from commercial intravenous presentation Prograf®.

Table S8. Median and percentiles of the scores obtained on the different items of the VFQ-25 scale at both visits.

	TE		TCD		
	**n**	**Md (Q1-Q3)**	**n**	**Md (Q1-Q3)**	**p-value**
**General health**	37	50 (25-75)	37	50 (25-75)	0.360
**Global Vision**	37	80 (60-80)	37	80 (60-80)	0.112
**Near vision**	37	83.3 (66.7-100)	37	91.7 (62.5-100)	0.520
**Distance vision**	37	91.7 (66.7-100)	37	95 (80.8-100)	0.152
**Social functioning**	37	100 (87.5-100)	37	100 (81.2-100)	0.325

**Role**	37	75 (56.2-100)	37	87.5 (68.7-100)	0.090
**Dependency**	37	100 (75-100)	37	100 (70.8-100)	0.874
**Mental health**	37	81.2 (56.2-87.5)	37	81.2 (65.6-87.5)	0.145
**Driving difficulties**	22	83.3 (50-91.7)	23	83.3 (50-91.7)	0.959
**Peripheral vision**	35	100 (50-100)	37	100 (75-100)	0.136
**Color vision**	37	100 (100-100)	37	100 (100-100)	0.206
**Ocular pain**	37	62.5 (56.2-87.5)	37	75 (62.5-87.5)	**0.004**
**Total score**	37	80,9 (65.7-89.7)	37	82.8 (76.6-92)	**0.003**

Md: median; Q1: 25th percentile; Q3: 75th percentile; n: population size. TCD: tacrolimus 0.015% in cyclodextrin; TE: tacrolimus 0.03% prepared from commercial intravenous presentation Prograf®

Table S9. Median and percentiles of total score of the VFQ-25 test obtained between visit 1 and 2 according to age groups and analysis of the correlation between age and the total score subscale.

		**TE**			**TCD**		
**Age**	**n**	**Md (Q1-Q3)**	**p-value**	**r**	**Md (Q1-Q3)**	**p-value**	**r**
<18	6	92.8 (89.9- 94.5)			93.9 (92.3- 95.3)		
18-65	22	80.9 (69.9- 86.8)	0.001	-0.647	81.6 (74.7- 91.4)	0.001	0.599
>65	8	68.5 (41.2-78.3)			77.7 (37.1-81.2)		

Md: median; n= population size (number of patients); Q1: 25th percentile; Q3: 75th percentil; r= correlation coefficient

Table S10. Median and percentiles of the scores obtained on the VAS of symptoms at both visits.

		**TE**		**TCD**		
		**Md (Q_1-Q_3)**	**n**	**Md (Q_1-Q_3)**	**n**	**p-value**
**Burning**	OD	5 (1.9-7)	38	2 (1-3.5)	33	**0.004**
	OS	5 (2-7)	32	2.5 (0.8-4.1)	30	**0.003**
**Discharge**	OD	1.5 (0-3)	39	1,5 (0-3)	34	0.345
	OS	2 (0-4.5)	33	1 (0-3)	30	0.130
**Lacrimation**	OD	2 (0.9-5)	38	1,5 (0.25-3)	33	0.308
	OS	1.5 (0.25-5.5)	33	1 (0-3)	30	0.100
**FBS**	OD	3 (0.4-5)	38	1,5 (0-3.5)	33	0.097
	OS	2 (0-5.3)	33	1,5 (0-4.3)	30	0.388
**Pain**	OD	1 (0-3.3)	38	1 (0-1.8)	33	0.303
	OS	1.3 (0-3)	32	0.5 (0-2)	30	**0.030**
**Photophobia**	OD	5 (2-7)	38	3 (1-6.8)	32	0.451
	OS	3 (1.3-7)	32	2 (0-6.8)	28	0.558

FBS: foreign body sensation; Md: median; OD: oculus dexter, right eye; OS: oculus sinister, left eye; Q1: 25th percentile; Q3: 75th percentile; n: population size. TCD: tacrolimus 0.015% in cyclodextrin; TE: tacrolimus 0.03% prepared from commercial intravenous presentation Prograf®
